# CREB3 Plays an Important Role in HPSE-Facilitated HSV-1 Release in Human Corneal Epithelial Cells

**DOI:** 10.3390/v14061171

**Published:** 2022-05-28

**Authors:** Tejabhiram Yadavalli, Pankaj Sharma, David Wu, Divya Kapoor, Deepak Shukla

**Affiliations:** 1 Department of Ophthalmology and Visual Sciences, University of Illinois at Chicago, Chicago, IL 60612, USA; yteja@uic.edu (T.Y.); sharmap@uic.edu (P.S.); dwu36@uic.edu (D.W.); dkapoo5@uic.edu (D.K.); 2 Department of Microbiology and Immunology, University of Illinois at Chicago, Chicago, IL 60612, USA

**Keywords:** herpes simplex virus, heparanase, CREB3, viral release, extracellular virus

## Abstract

Herpes simplex virus type-1 (HSV-1) exploits several host factors to enhance its replication and release from infected cells. It induces the production of host enzyme heparanase (HPSE) to aid in egress. While the mechanism by which HPSE assists in viral release is well-characterized, other host factors that are recruited along with HPSE for viral release are less well understood. In this study, we identify cyclic-AMP-responsive element-binding protein3 (CREB3) as a key player in HPSE-facilitated HSV-1 egress. When CREB3 is transiently upregulated in human corneal epithelial cells, HSV-1 release from the infected cells is correspondingly enhanced. This activity is linked to HPSE expression such that HPSE-transfected corneal epithelial (HCE) cells more highly express CREB3 than wild-type cells while the cells knocked out for HPSE show very little CREB3 expression. CREB3-transfected HCE cells showed significantly higher export of HPSE upon infection than wild-type cells. Our data suggests that coat protein complex II (COPII), which mediates HPSE trafficking, is also upregulated via a CREB3-dependent pathway during HSV-1 infection. Finally, the co-transfection of CREB3 and HPSE in HCE cells shows the highest viral release compared to either treatment alone, establishing CREB3 as a key player in HPSE-facilitated HSV-1 egress.

## 1. Introduction

Herpes simplex virus type-1 (HSV-1) is a prototypic member of the herpesvirus family of DNA viruses. HSV-1 infection may lead to mucocutaneous and ocular lesions in healthy patients. More serious consequences of HSV-1 infection are reported in immunocompromised patients. This virus is highly transmissible and seropositivity can be as high as 80% in different areas of the world [1]. Ocular morbidity caused by HSV-1 is considered a leading cause of infection-associated blindness in developed countries [2]. It can enter virtually all human cell types, and as a prime example of a successful pathogen [3], it can exploit an array of host molecules to help with its lifecycle, including its egress from a host cell. Heparanase (HPSE), a host enzyme that participates in viral egress, is the only endoglycosidase known to cleave heparan sulfate (HS), a polysaccharide found on the cell surface [4]. HPSE was first identified due to its important role in tumor metastasis. It cleaves HS chains to facilitate the migration of tumor cells through the vasculature [5]. Recently, its roles in virology were also elucidated, exemplified by its function in HSV-1 release and pathogenesis. Late during infection, HPSE is transported to the cell surface to cleave off HS moieties, which are commonly used by HSV-1 and other viruses for attachment to cells. By removing HS, it facilitates the egress and release of viral progenies [6,7]. Since HS is heavily expressed in the extracellular matrix, its enzymatic removal by HPSE ensures a more efficient release of newly generated virions. For this reason, it has been proposed that HSV-1 uses HPSE as a functional switch that enables its transition from an ‘attachment’ mode during entry to a ‘detachment’ mode during viral egress [6]. HPSE also directly induces its own upregulation via the NF-κB signaling pathway [7,8]. In addition, HPSE plays a role in promoting the shedding of heparan sulfate proteoglycan syndecan-1, connecting it even more strongly with extracellular viral release as a ‘master regulator’ [8]. When HPSE is knocked down, infectivity is significantly impaired for HSV-1 and HSV-2 [6,9]. HPSE is also a pro-survival factor: a complete knockdown of HPSE ultimately results in cell death, making it difficult to directly target HPSE for viral therapy [10].

Cyclic AMP-responsive element-binding protein 3 (CREB3) is a transcription factor that resides in the endoplasmic reticulum (ER) [11]. In the unfolded protein response to ER stress, it induces the expression of Herp, an organizer of the ER-associated degradation (ERAD) complex [12,13]. With the ties of HSV-1 to the UPR, which it disarms in order to continue producing its own proteins, HSV-1 has long been suspected to modulate CREB3 during infection [14,15]. However, it was not until recently that the role of CREB3 in HSV infection was specified. Among the ER-resident regulators of ER stress, only CREB3 is upregulated during HSV-1 infection [2]. When CREB3 is silenced using an siRNA, a loss in host survival and viral replication was noted, which subsequently resulted in decreased viral load [2]. In contrast, overexpression of CREB3 was pro-survival, with no significant effect on infectivity, but resulted in a low but significant increase in the amount of extracellular-secreted virus during HSV-1 infection [2].

Quite interestingly, an upregulation of either HPSE or CREB3 expression results in improved cell survival while their knockdown results in decreased cell survival and viral replication [16,17] Despite the similarities and an apparent positive correlation observed between HPSE and CREB3 expression, especially during the detachment phase of HSV-1 infection, very little is known about the significance of CREB3 in viral release. In this study, our objective was to find a novel role for CREB3 in HSV release and link it to the key host enzyme HPSE. To test our hypothesis, we used transient transfection of CREB3 and HPSE plasmids to measure the extracellular viral load, expression of HPSE and CREB3 proteins via Western blotting, COPII vesicle protein mRNA transcript measurement via qPCR, and extracellular protein release via slot-blot techniques.

## 2. Materials and Methods

### 2.1. Cells and Viruses

HCE cells (RCB1834 HCE-T) were obtained from Kozaburo Hayashi (National Eye Institute, Bethesda, MD, USA) and cultured in minimum essential medium (MEM) (Life Technologies, Carlsbad, CA, USA) supplemented with 1% penicillin-streptomycin (P/S) (Life Technologies, Carlsbad, CA, USA) and 10% fetal bovine serum (FBS) (Sigma-Aldrich, St. Louis, MO, USA). African green monkey kidney (Vero) cells were provided by P. G. Spear (Northwestern University, Chicago, IL, USA) and cultured in Dulbecco’s modified Eagle’s medium (DMEM; Life Technologies, Carlsbad, CA, USA) supplemented with 1% P/S and 10% FBS. Cells were cultured in a Heracell VIOS 160i CO_2_ incubator (Thermo Fisher Scientific Pittsburgh, PA, USA). HPSE wild-type and knock-out mouse embryonic fibroblasts were generously provided by Dr. Israel Vlodavsky (Rappaport Institute, Haifa, Israel) [18]. 

HSV-1 (KOS K26RFP) was obtained from Dr. P. Desai (Johns Hopkins University, Baltimore, MD, USA). Viral stock was generated on Vero cells and stored at −80 °C. All infections were performed at a 0.1 multiplicity of infection (MOI).

### 2.2. Transfection

Upregulation of CREB3 and HPSE was accomplished using either plasmid containing CREB3-triple-FLAG tag (p3XFlag-LZIP) (Addgene, Watertown MA, USA) or human HPSE (pIRES2 EGFP-HPSE1) (Dr. Ralph Sanderson, University of Alabama at Birmingham, Birmingham, AL, USA). All transfections were executed using Lipofectamine-2000 transfection reagent (Life Technologies) according to the manufacturer’s specifications and incubated with cells for a minimum of 24 h in Opti-MEM (Life Technologies, Pittsburgh, PA, USA) prior to experimentation. Transfection efficiencies ranged from 50–60%.

### 2.3. Immunoblotting

Cells were collected in Hanks’ Balanced Salt Solution (Life Technologies) after a minimum incubation period of 10 min. The cells were then spun down at 800 g for 5 min and the Hanks’ aspirated and replaced with 100 μL of 1:1 radioimmunoprecipitation assay (Millipore Sigma) buffer with protease phosphatase inhibitor cocktail (Halt; Thermo Fisher Scientific) for a 30 min incubation on ice. Whole-cell protein extracts were then collected after a 15 min centrifuge at 13,500 rpm at 4 °C. NuPAGE LDS Sample Buffer (Invitrogen, NP00007, Waltham, MA, USA) and β-mercaptoethanol (Thermo Fisher Scientific Pittsburgh, PA, USA) were added to the extracts and heated at 90 °C for 9 min. After the samples were cooled to room temperature, the protein contents were added in equal amounts to 4 to 12% SDS-polyacrylamide gel electrophoresis loading gels and run at a constant voltage of 70 V for 3 h. At this point, the gels were transferred to a nitrocellulose membrane (IB23001, Invitrogen, Waltham, MA, USA) using an iBlot 2 dry transfer machine (Thermo Fisher Scientific, Pittsburg, PA, USA). The membranes were blocked in 5% skim milk (Difco) in tris-buffered saline (TBS; Thermo Fisher Scientific, Pittsburg, PA, USA) with 0.1% Tween 20 (Sigma-Aldrich, Burlington, MA, USA) (TBST) for 1 h at room temperature. Following the blocking procedure, membranes were transferred into the primary antibodies at 1:1000 in milk for an overnight incubation at 4 °C. The next day, the blots were washed 3 times for 5 min with TBST and transferred into secondary immunoglobulin G antibody (Jackson ImmunoResearch Laboratories, West Grove, PA, USA) diluted 1:10,000 in 5% skim milk at room temperature for 1 h. Bands were imaged on an ImageQuant LAS 4000 imager (GE Healthcare Life Sciences, Chicago, IL, USA) using SuperSignal West Pico maximum sensitivity substrate (Pierce, 34080).

Anti-glyceraldehyde-3-phosphate dehydrogenase (GAPDH) (ProteinTech, Rosemont, IL, USA, 10494-1-AP) was used as a loading control. Anti-HSV-1 VP16 mouse monoclonal antibody (Santa Cruz Biotechnology, sc-7545) and anti–HSV-1 gB mouse monoclonal antibody (Abcam, 6506) were used as proxies to determine the amount of viral infection. Luman/CREB3 antibody was obtained from ProteinTech (11275-1-AP), HPSE antibody was obtained from Advanced Targeting Systems, San Diego, CA, USA (HP130), GRP78 (C50B12 #3177) was obtained from cell signaling technologies, USA, and syndecan-1 was obtained from Santa Cruz Biotechnology, Santa Cruz, CA, USA (sc-6532).

### 2.4. Plaque Assay

Monolayers of HCE cells were infected with HSV-1 (KOS RFP 0.1 MOI) in Opti-MEM (Thermo Fisher Scientific, Pittsburgh, PA, USA). After 2 h of incubation at 37 °C, 5% CO_2_, the virus-containing media was aspirated and replaced with MEM supplemented with 1% P/S and 10% FBS as above. Then, 24 h post infection, infected cells and their media were collected separately and the cells were resuspended in 500 μL of Opti-MEM. The samples were then sonicated with a probe sonication system at 70% amplitude for 30 s. Lysates or media containing egressed virus were used as inoculants for the plaque assay. Fully confluent Vero cells were washed with PBS and inoculated for 2 h at 37 °C, 5% CO_2_ at multiple dilutions. The cells were then washed with PBS and overlaid for 72 h with DMEM whole media with 0.5% methylcellulose (Sigma-Aldrich, Burlington, MA, USA). After the incubation period, the cells were fixed with methanol and stained with crystal violet for quantification.

### 2.5. Slot Blot 

Supernatants of infected HCE cells were vacuum filtered through an Immobilon-Ny+ nylon membrane (Millipore Corporation, Billerica, MA, USA) using the Bio-Dot SF apparatus (Bio-Rad, Hercules, CA, USA). The membranes were then blocked overnight with antibodies specific for CREB3 (ProteinTech 11275-1-AP), HSV-1 gB (Abcam, 6506), syndecan-1 (Santa Cruz Biotechnology, sc-6532), and HPSE (Advanced Targeting Systems, HP130) followed by the corresponding species-specific secondary antibodies. Detection was accomplished with the ImageQuant LAS 4000 imager (GE Healthcare Life Sciences) as above.

### 2.6. Quantitative PCR

TRIzol (Life Technologies) was used to extract RNA from cells according to the manufacturer’s directions. RNA was then transcribed to cDNA using a High-Capacity cDNA reverse transcription kit (Applied Biosystems, Foster City, CA, USA). Real-time qPCR was conducted with a Fast SYBR green master mix (Applied Biosystems) employing QuantStudio 7 Flex (Applied Biosystems). All the primers used in this manuscript were pre-designed and pre-validated from Millipore Sigma Aldrich (KiCqStart^®^ SYBR^®^ Green Primers).

## 3. Results

### 3.1. CREB3 Upregulation Potentiates HSV-1 Egress

**Figure 1:**
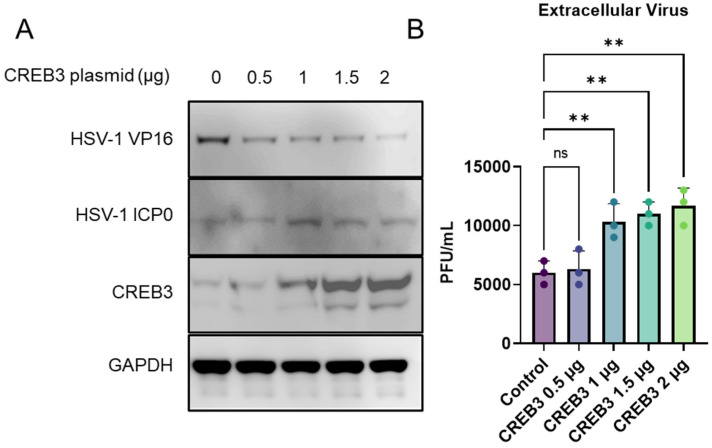
Upregulation of CREB3 causes an increase in extracellular virus: Human corneal epithelial cells were transfected with a control plasmid or increasing concentrations of CREB3 plasmid for 24 h followed by infection with HSV-1 at 0.1 MOI. At 24 h post infection, samples were collected for analysis. (**A**) Representative immunoblot images confirming the upregulation of CREB3 protein with increasing plasmid concentrations (**B**) Cellular supernatants were collected and centrifuged to remove any cellular components prior to overlaying on Vero cells to titrate extracellular infectious virion particles. One-way ANOVA was performed between the datasets to determine statistical significance. ** *p*-value < 0.01.

In a recent study, we observed a slight but significant increase in the amount of extracellular virus, with no significant changes to intracellular virus titers when CREB3 was overexpressed [2]. To study this phenomenon further, we decided to overexpress CREB3 in human corneal epithelial (HCE) cells, a natural target cell line. HCE cells were transfected with a CREB3 plasmid at increasing plasmid concentrations (0.5, 1, 1.5, and 2 μg) overnight in a serum-free culture media (opti-MEM). After incubation, the cells were infected with HSV-1 for 24 h and then processed for immunoblotting. The cell and culture supernatants were collected for the plaque assay. Immunoblotting of the cells confirmed a successful upregulation of CREB3, which increased with increasing concentrations of plasmid (Figure 1A). In parallel, the plaque assays from the culture supernatants demonstrated a corresponding increase in the extracellular or released virus content. It was clear that with an increase in the expression of CREB3, there was a corresponding dose-dependent increase in the extracellular virus release (Figure 1B); however, no difference in the intracellular viral load was observed. 

### 3.2. An Overexpression of HPSE Leads to an Increase in CREB3, but CREB3 Overexpression Does Not Cause Higher HPSE Expression

**Figure 2:**
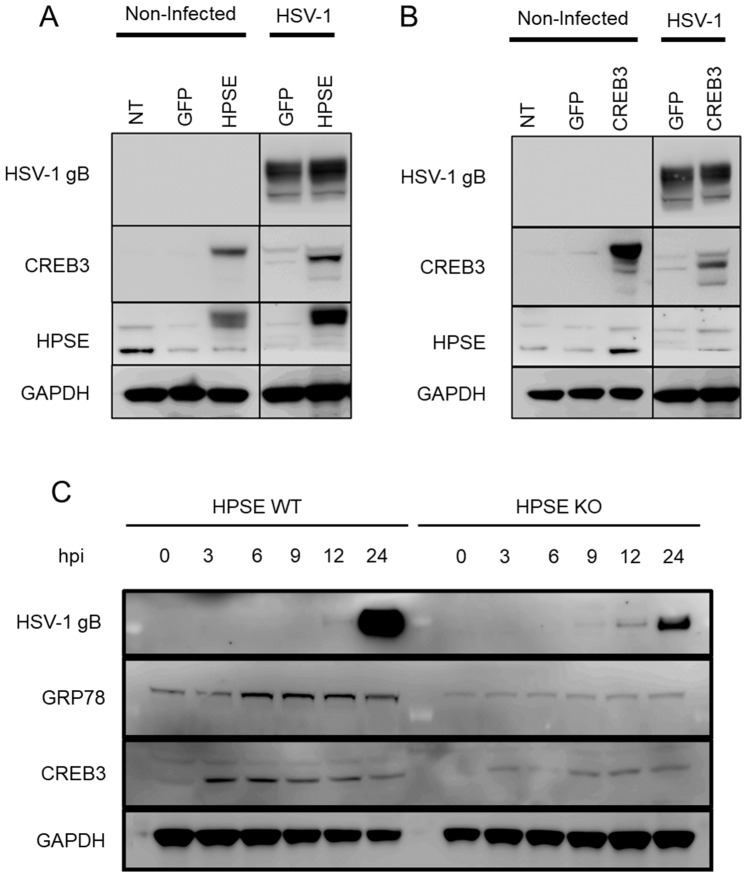
Upregulation of HPSE increases CREB3 expression: (**A**) Human corneal epithelial cells were transfected with a control plasmid, HPSE-GS3 (constitutively active form of HPSE), or full-length HPSE plasmid for 24 h followed by infection with HSV-1 at 0.1 MOI. At 24 h post infection, samples were collected for immunoblotting analysis. (**B**) Human corneal epithelial cells were transfected with a control plasmid or CREB3 plasmid for 24 h followed by infection with HSV-1 at 0.1 MOI. At 24 h post infection, samples were collected for immunoblotting analysis. (**C**) Heparanase wild-type or knockout mouse embryonic fibroblasts were infected with 0.1 MOI HSV-1 and cell lysates were collected at the indicated time points for immunoblotting analysis.

After confirming that CREB3 expression was correlated with viral egress, it was important to determine a tentative mechanism for our findings. Based on the strong correlative aspects between HPSE and CREB3 during HSV-1 infection, we hypothesized that HPSE and CREB3 overexpression might correspondingly upregulate each other during viral infection. To test this hypothesis, HCE cells were transfected with either a GFP plasmid or a full-length HPSE plasmid overnight followed by infection with HSV-1 for 24 h. The following day, samples were collected for immunoblotting analysis. In comparison to the GFP-transfected cells, the HPSE-transfected cells showed greater expression of CREB3, both in the infected and non-infected groups (Figure 2A). Conversely, a separate set of HCE cells were treated with Lipofectamine-2000 alone (NT group) or transfected with a GFP plasmid or a CREB3 plasmid for 24 h followed by 24 h of HSV-1 infection. There was no appreciable difference in the HPSE expression between the GFP- and CREB3-transfected groups (Figure 2B). As anticipated, the infected group showed greater levels of HPSE than the non-infected group due to viral induction. Lastly, to check whether CREB3 expression would decrease with HPSE, we used an HPSE knockout model of mouse embryonic fibroblasts and infected them with HSV-1. Cell lysates were collected at different times post infection for immunoblotting analysis. While the expression at 0 h in both HPSE wild-type and knockout cell types was very low, CREB3 expression increased immediately post infection in both cell types. However, the blots revealed a marked decrease in the relative expression of CREB3 and the unfolded protein response (UPR) regulator, GRP78, in HPSE knockout cells compared to wild-type cells at 3, 6, 9, 12, and 24 h post-infection (Figure 2C). The lower expression of both GRP78 and CREB3 in knockout cells could indicate a lower response to stress, either due to a decreased viral load or decreased HPSE activity.

### 3.3. CREB3-Transfected Cells Export Greater Amounts of HPSE

**Figure 3:**
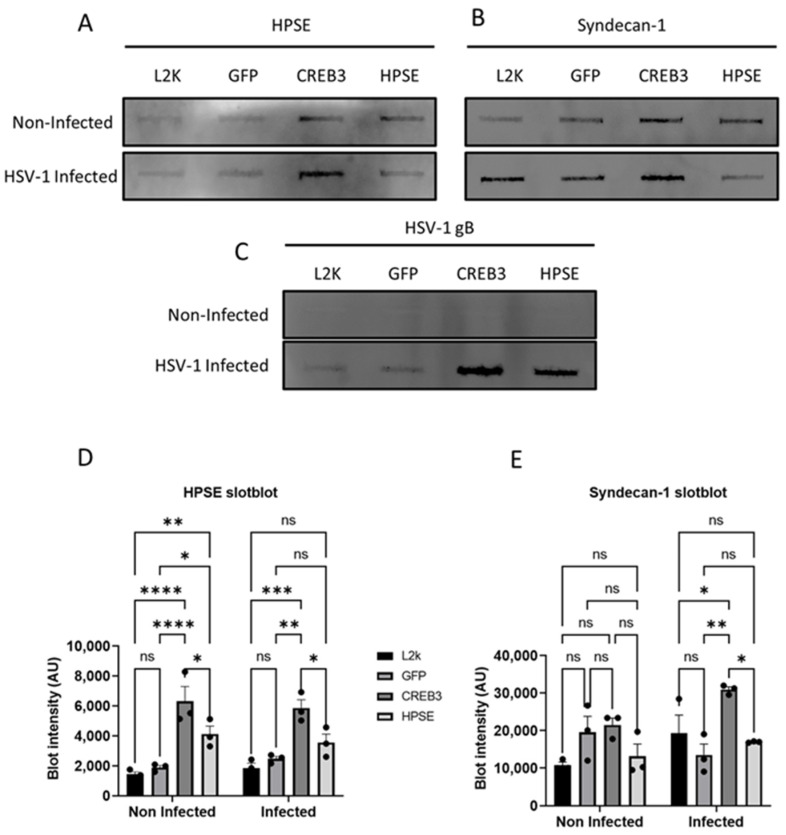
Upregulation of CREB3 increases extracellular HPSE: Human corneal epithelial cells were not transfected (lipofectamine only—L2K) or transfected with a control plasmid, CREB3 plasmid, or full-length HPSE plasmid for 48 h with or without HSV-1 infection. Cellular supernatants were collected and analyzed via a slot blot technique. (**A**) HPSE secretion, (**B**) Syndecan-1 secretion, and (**C**) HSV-1 envelope glycoprotein-B secretion were detected using the slot blot technique. (**D**,**E**) Experiments mentioned in (**A**,**B**) were performed in triplicate and the quantification of the slot blot bands was performed using image J and represented as bar charts. * *p*-value < 0.05, ** *p* < 0.01, *** *p* < 0.001, **** *p* < 0.0001.

Given that CREB3 is upregulated with HPSE overexpression but not vice versa, we hypothesized that CREB3 expression in some way is able to assist HPSE functionality. CREB3 is a transcription factor known for its role in upregulation of proteins that are important for ERAD and COPII vesicle transport [19,20]. Through this, CREB3 assists in either the degradation of misfolded proteins or post-translational modification and secretion of folded proteins. Based on this knowledge, we hypothesized that CREB3 may be helping HPSE traverse the ER to Golgi at an improved rate, allowing it to be secreted at the cell surface. To investigate this link between CREB3 and HPSE, HCE cells were either treated with Lipofectamine-2000 alone or transfected with a control plasmid, CREB3 plasmid, or full-length HPSE plasmid for 48 h with or without HSV-1 infection. The supernatants of the monolayers were collected for slot blot analysis. With the upregulation of HPSE, we observed that CREB3 was not appreciably upregulated in the extracellular space but remained unaffected (Figure 3A). For the HPSE-transfected group, it was also noted that the HSV-1 gB and syndecan-1 shedding content was observed to be mildly higher in the supernatant, but this was not significant (Figure 3B,C). On the other hand, CREB3-transfected cells showed increased secretion of HPSE in the supernatants when compared to control transfected cells. Furthermore, in every treatment group, the HPSE content was much greater during infection than without (Figure 3D). Finally, as an indicator of the extracellular virus content, the HSV-1 gB levels were elevated in the CREB3 upregulated group (Figure 3E). Syndecan-1 shedding, an indicator of increased viral release and heparanase levels, was also elevated in non-infected and infected cells [21]. 

### 3.4. Upregulation of CREB3 but Not HPSE Causes an Increase in COPII-Associated Transcripts

**Figure 4:**
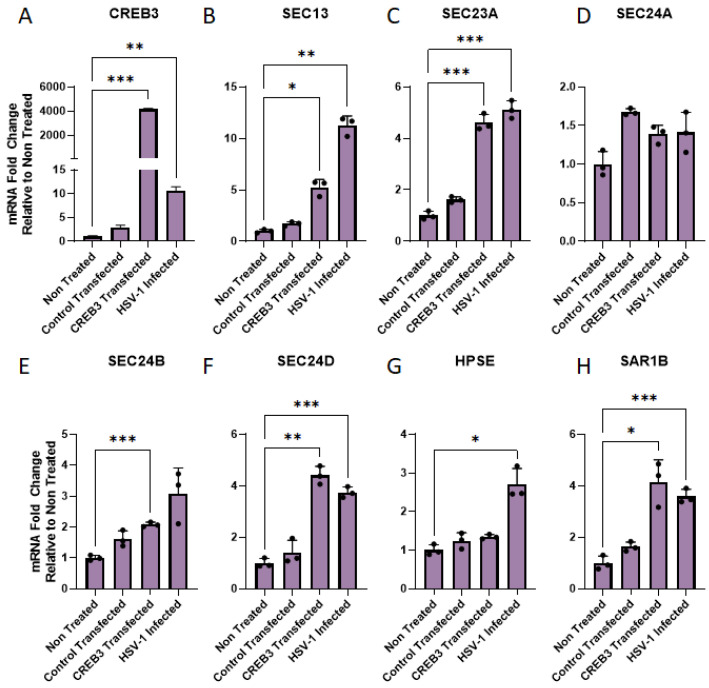
Upregulation of CREB3 increases COPII vesicle transcripts: (**A**) Human corneal epithelial cells were transfected with a control plasmid, CREB3 plasmid, or full-length HPSE plasmid for 48 h. Total cellular RNA was extracted from the samples using the TriZol technique, reverse transcribed to form cDNA, and analyzed via RT-qPCR. (**A**) qPCR confirmation of CREB3 upregulation in transfected cells. (**B**–**H**) COPII vesicle machinery and HPSE mRNA transcripts shown as a fold-change relative to non-transfected samples. Two-way ANOVA was used to determine statistical differences between rows (treatment groups) within a given a column (mRNA transcript expression level). * *p*-value < 0.05, ** *p* < 0.01, *** *p* < 0.001.

Given that our hypothesis that CREB3 facilitates improved HPSE secretion into the extracellular space, we wanted to confirm whether this was in effect a consequence of increased COPII vesicle protein upregulation. There are multiple components that constitute the COPII vesicle machinery. Many of these proteins are upregulated during HSV-1 infection [22]. In order to ascertain whether the upregulation of CREB3 resulted in greater trafficking of proteins to the cell surface, we evaluated the mRNA transcripts of SEC13, SEC23A, SEC24A, SEC24B, SEC24D, and SAR1B during CREB3 overexpression (Figure 4A). Our results indicate that with CREB3 overexpression or HSV-1 infection, SEC13 (Figure 4B), SEC23A (Figure 4C), SEC24B (Figure 4E), SEC24D (Figure 4F), and SAR1B (Figure 4H) were significantly upregulated. However, SEC24A (Figure 4D) remained unchanged with CREB3 overexpression and HSV-1 infection. Additionally, HPSE expression remained unchanged with CREB3 overexpression; however, it significantly increased with viral infection.

### 3.5. The Co-Expression of CREB3 and HPSE Increases Viral Egress

**Figure 5:**
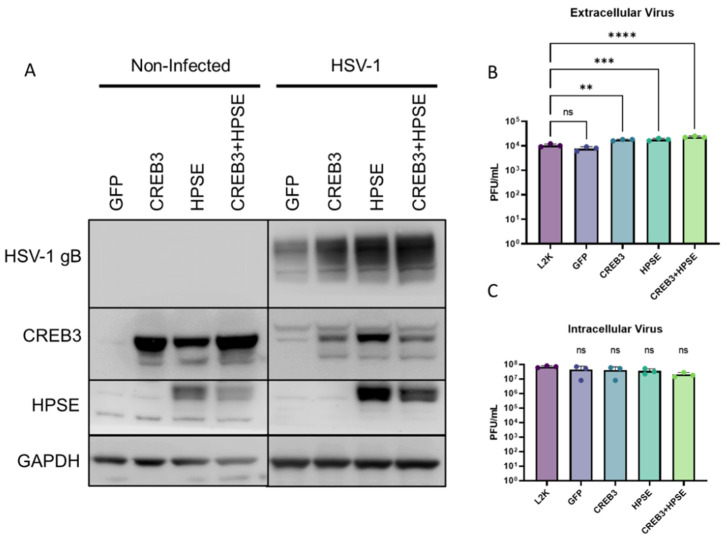
Co-expression of CREB3 and HPSE increased extracellular virus release: (**A**) Human corneal epithelial cells were left non-transfected, treated with lipofectamine alone, or transfected with a control plasmid, CREB3 plasmid, or full-length HPSE plasmid for 24 h followed by infection with HSV-1 for 24 h. (**A**) Cell lysates were immunoblotted to confirm co-transfection of CREB3 and HPSE. (**B**) Cellular supernatants were collected, centrifuged, and overlayed on Vero cells to titrate infectious titer present in the extracellular space via a plaque assay. (**C**) Cell lysates were collected, ultrasonicated, and overlaid on Vero cells to titrate intracellular infectious titer via a plaque assay. One-way ANOVA was performed between the sample sets to determine statistically significant differences. ns-non-significant ** *p*-value < 0.01, *** *p* < 0.001, **** *p* < 0.0001.

After determining that CREB3 was associated with COPII-vesicle formation, we wanted to assess whether the co-expression of CREB3 and HPSE would increase viral egress beyond either group alone. HCEs were thus either treated with Lipofectamine 2000 alone, or transfected with a GFP plasmid, CREB3 plasmid, or full-length HPSE plasmid followed by HSV-1 infection. The cells lysates and supernatants were collected at that time for separate processing. Lysates were processed for immunoblotting to confirm successful HPSE and CREB3 co-transfection (Figure 5A). Cellular supernatants were then processed and evaluated for their viral content with the plaque assay. It was revealed that CREB3 and HPSE co-transfected cells had greater viral egress than CREB3 and HPSE alone, which had greater viral egress than either the mock GFP plasmid- or Lipofectamine 2000-treated groups (Figure 5B). By contrast, there was no appreciable difference in the intracellular virus in these groups after sonication of the samples and overlay onto Vero cells (Figure 5C).

## 4. Discussion

Our study is the first to report a role for CREB3 in HPSE-facilitated HSV-1 release. We showed that CREB3 might facilitate the secretion of HPSE and a corresponding increase in Syndecan-1 shedding, which is a marker of HSV-1 release from infected cells [21]. A role for CREB3 in HSV-1 release is further supported by our observation that a combinatorial upregulation of HPSE and CREB3 expression resulted in a significantly increased release of HSV-1 from the host cells. Another interesting aspect that was observed during this study was the presence of differential mobility bands in the Western blots of CREB3, specifically during HSV-1 infection. While the underlying reason for this observation is worth further investigation, it is out of the scope of this study. We also found evidence that CREB3, through the upregulation of SEC13, SEC23, SEC24, and SAR1, which are COPII vesicle proteins, allows for increased secretion of HPSE and Syndecan-1 shedding. It is already well established that higher HPSE and Syndecan-1 shedding leads to higher HSV-1 release [21]. In addition, for the first time, we demonstrated a unique correlation in the expression of HPSE and CREB3. We showed that while HPSE upregulation results in the overexpression of CREB3, the reverse was not found. Our data show that both CREB3 and HPSE, which are pro-survival proteins, help in viral release from an HSV-1-infected cell and their expression may be inter-dependent, especially during HSV-1 infection. CREB3, a host transcription factor, has been shown by our group to be upregulated upon HSV-1 infection [2]. Previous reports have shown that CREB3 transcription is in turn mediated by NF-κB [20]. It is well-known that HSV-1 infection results in the activation and nuclear translocation of NF-κB [23], which could, in turn, upregulate CREB3 transcription. CREB3, such as ATF6, translocates from the ER to the Golgi, where it is activated via regulated intramembrane proteolysis (Rip) prior to translocating to the nucleus [24]. While in the nucleus, CREB3 has been shown to modulate several proteins, including those associated with ER-associated degradation, COPII vesicle formation, cell survival, etc. [19,20]. We and others have previously shown that COPB1, a coatomer complex protein that helps in protein transport from the ER to the Golgi and trans-Golgi-network for secretion, is upregulated due to CREB3 activity. Due to these functionalities, CREB3 might be among the only UPR-associated proteins that HSV-1 does not disarm. HPSE, prior to its secretion, is folded in the ER, glycosylated, and undergoes post-translational modifications in the trans-Golgi-network [25]. This might be the reason why HPSE upregulation indirectly triggers the upregulation of CREB3 via NF-κB activation. It is interesting, however, that CREB3 is upregulated due to HPSE overexpression only during HSV-1 infection. While it is difficult to speculate whether this is a pro-viral or anti-viral mechanism, it results in the secretion of excess virion particles from an infected cell. Previous studies have shown that viral infection results in the increase in COPII vesicle machinery proteins that help transport viral glycoproteins to the plasma membrane [26]. Genetic upregulation of CREB3, specifically in non-infected cells, increased COPII vesicle proteins, such as Sec13, Sec23, and SAR1, which might be an indication that CREB3 is able to increase the secretory potential of the cell. To check whether this indeed is true, we performed a slot blot experiment with the extracellular supernatants of the cells, which were transfected with CREB3 or HPSE. To our delight, this was indeed true. We not only saw an increase in the amount of HPSE in the extracellular secreted space but also HSV-1 glycoprotein-B and Syndecan-1. We have previously observed that Syndecan-1 shedding is upregulated upon HSV-1 infection, which is mediated by MMP3 and 7 and triggered by HPSE upregulation [8]. Even in non-infected cells, CREB3 upregulation increased the amount of HPSE in the extracellular space, which could indirectly have caused a greater amount of Syndecan-1 shedding. This shedding was even higher in case of infected cells. Hence, CREB3 overexpression alone also might be able to trigger excessive viral egress without HPSE activity, which can be determined by titrating extracellular viral particles in an HPSE knockout cell line. Furthermore, the increase in COPII vesicle machinery proteins might be a result of increased CREB3 expression, which needs to be transported to the Golgi and has nothing to do with viral egress. This can be determined by staining cells at different times post infection for COPII proteins, viral proteins, and CREB3. However, these experiments do not form the basis for our current study and will be considered in the future. Finally, we hypothesized that if CREB3 improved HPSE secretion, which indirectly results in the release of extracellular virus, then a combinatorial upregulation of CREB3 and HPSE could further increase viral release from the cell. While a higher amount of viral release was observed in cells transfected with CREB3 or HPSE alone, their combinatorial transfection further increased the amount of viral release from the cells. Here, the upregulation of CREB3 alongside HPSE could be helping the excess amount of HPSE to be secreted from the cell, which in turn is cleaving Heparan sulfate, and promoting Syndecan-1 shedding, all of which are increasing viral release from the infected cell surface.

Herpesviruses exploit the host cell machinery for a plethora of functions, including entry, replication, assembly, and egress. Herpesviruses, in general, prefer to spread from one cell to another intracellularly rather than egressing from the host cell [27]. This ensures that the virus is less exposed to the hostile immune environment surrounding the cell. While the formation of syncytia allows the virus to infect a neighboring cell, the formation of large syncytia can also attract immune cells to the site of infection [28]. In this regard, both viruses and host cells have developed sophisticated strategies and counter strategies that are advantageous to each of them. In our current study, we observed that upregulation of CREB3 or HPSE or both results in the extracellular release of the virus from an infected cell. However, it is hard to decipher whether this is advantageous or disadvantageous to the virus/host. This might be advantageous to the virus because the virus is set free from the cellular microenvironment and is able to travel to other cells, where it might be able to infect non-neighboring cells. It may be disadvantageous to the virus because the virus/viral proteins are secreted from the cell, making them more susceptible to host immune recognition (advantageous to the host) or disadvantageous to the host because an increase in viral egress and immune reaction might spark irrecoverable damage to the infected tissue site. We have previously shown that viral activation of HPSE can indeed spark a cascade of immune reactions, which result in severe damage to the host tissue [7]. While our study has made several interesting observations, the limitations of our study include the absence of physiological relevance associated with our observation. Our study does not address the question whether a simultaneous upregulation of HPSE and CREB3 could result in a pro-host or pro-viral outcome in an animal model of HSV-1 infection. Limitations of this study also include the inability to explain our observation about how or why CREB3 is secreted from host cell and what function it has. All these interesting observations make way for future studies, which might be able to answer them in further detail.

## Data Availability

Not applicable.
